# Anatomical study of pterygoid implants: artery and nerve passage through bone dehiscence of the greater palatine canal

**DOI:** 10.1186/s40729-024-00560-z

**Published:** 2024-11-07

**Authors:** Shuichiro Taniguchi, Masahito Yamamoto, Tomohito Tanaka, Tianyi Yang, Genji Watanabe, Yuki Sugiyama, Takahiro Takagi, Gen Murakami, Shogo Hayashi, Shinichi Abe

**Affiliations:** 1 Department of Anatomy, Tokyo Dental College, 2-9-18 Kandamisaki-Cho, Chiyoda-Ku, Tokyo, 101-0061 Japan; 2 Department of Anatomy, Division of Basic Medical Science, Tokai University School of Medicine, 143 Shimokasuya, Isehara-Shi, Kanagawa, 259-1193 Japan; 3 Division of Internal Medicine, Iwamizawa Koujinkai Hospital, 297 Shimon-Cho, Iwamizawa, 068-0833 Japan

**Keywords:** Pterygoid implant, Greater palatine canal, Bone dehiscence, Greater palatine nerve, Descending palatine artery

## Abstract

**Purpose:**

Pterygoid implants are an alternative approach to avoid sinus-lifting or other grafting procedures. During pterygoid implant placement, dental surgeons risk damaging the greater palatine canal (GPC). However, they do not have sufficient reasons to avoid GPC injury. This study performed a detailed morphological analysis of the GPC to determine susceptibility to damage during pterygoid implant surgery.

**Methods:**

To understand the detailed morphology of the GPC, gross anatomical analysis, histological analysis, and bone morphometry via micro-computed tomography were performed.

**Results:**

We found that the medial wall of the GPC communicated with the nasal cavity through the bone dehiscence. The dehiscence appeared near the inferior nasal concha in 72.4% of the cadavers. The nerve and artery passed from the GPC to the nasal mucous membrane through the dehiscence. Given that the greater palatine nerve passed medial to the descending palatine artery in the GPC, the descending palatine artery is damaged first rather than the greater palatine nerve during pterygoid implant surgery.

**Conclusions:**

Dental surgeons who penetrate the GPC using an implant body may extend the bleeding to the nasal mucosa, which seems to spread the inflammation to the nasal cavity.

## Background

Pterygoid implants, first proposed by Tulasne in 1992 [1], have been used an alternative approach to avoid sinus-lifting or other grafting procedures during the treatment of the posterior maxilla [2–8]. Pterygoid implants target the anatomical structures of the posterior maxilla, including the maxillary tuberosity, the pyramidal process of the palatine bone, and the pterygoid process of the sphenoid bone [9]. Given that the pyramidal process and pterygoid process have greater bone quality than do the maxillary alveolar process and tuberosity [3], the posterior maxillary region has been considered useful for dental implants. Moreover, studies have shown that pterygoid implants have high survival rates, comparable to those observed with conventional implant techniques [2–8]. However, the placement of pterygoid implants is extremely difficult and requires dental surgeons to accumulate a considerable amount of surgical experience [6]. Moreover, pterygoid implant placement requires surgeons to have thorough knowledge of anatomical structures [2–9], particularly regarding the internal maxillary artery, the maxillary sinus, the greater palatine canal (GPC), the posterior superior alveolar nerve, and pterygoid muscles [3, 9, 10].

Pterygoid implant placement has been associated with the risk of damaging the GPC [11], a thin passage through which the descending palatine artery, vein, and greater palatine nerves run to supply or innervate the mucosa on the maxillary and palatine bones via the greater palatine foramen (GPF) [12]. The GPC is formed medially by the perpendicular plate of the palatine, anteriorly by the infratemporal surface of the maxilla, and posteriorly by the pterygoid process of the sphenoid [13]. Moreover, the GPC runs posteriorly to the maxillary sinus, with the distance from its posterior wall to the GPC being unique for every person [7]. On the other hand, the nasal cavity is located just medial to the GPC [12]. During cadaver dissection, the GPC can be identified by removing the mucosa of the posterior part of the nasal cavity [12]. Interestingly, a radioanatomic study using computed tomography (CT) showed that 38% of cadavers showed dehiscence in some parts of the medial bony wall of the GPC toward the nasal cavity [14]. However, dental surgeons do not have sufficient reasons to avoid GPC injury during pterygoid implants. The current study performed a detailed morphological analysis of the GPC to determine susceptibility to damage during pterygoid implant surgery.

## Materials and methods

This study was performed in accordance with the provisions of the Declaration of Helsinki 1995 (as revised in Edinburgh 2013). We used cadavers and skulls donated to Tokyo Dental College for research and education on human anatomy after obtaining approval from the Tokyo Dental College ethics committee (No. 922-2). In all cases, the cause of death was ischemic heart or brain disease, and no macroscopic pathology was evident in the head, thorax, or abdomen were noted during dissection to obtain the specimens.

After removing the brain, the cadaveric head was divided into the left and right halves. Thereafter, we dissected the both adult nasal cavities in 5 donated cadavers (2 males and 3 females aged 72–88 years at death). To obtain histological sections, we removed the GPC and its surrounding structures in 10 donated cadavers (6 males and 4 females aged 71–92 years at death). After removing the brain, the cadaveric head was divided into the left and right halves. The entire mandible was then removed along with the masseter and medial pterygoid muscles. A horizontal cut was made on the upper side of the maxillary sinus to obtain an almost cubic block of the GPC. The cadavers had been fixed via arterial perfusion of 10% v/v formalin solution and stored in 50% v/v ethanol solution for over 3 months. GPC specimens were decalcified by incubating them in Plank–Rychlo solution (AlCl_2_/6H_2_O, 7.0 w/v%; HCl, 3.6; HCOOH, 4.6) at room temperature for 1–2 weeks. After routine procedures for paraffin embedding [15–19], we prepared large horizontal and frontal sections at 200-µm intervals. All histological images were obtained using a Nikon Eclipse 80.

We removed the GPC and its surroundings from 29 cadavers (16 males and 13 females aged 68–92 years at death) following a histological study. Samples were scanned using a micro-CT system (HMX-225 Actis4; Tesco Co., Tokyo, Japan). Imaging conditions included a tube voltage of 100 kV, tube current of 70 µA, magnification of 2.5 × , slice width of 5 µm, matrix size of 512 × 512, slice pitch of 50 µm, and voxel size of 140 × 140 × 50 µm. The imaging intensifier was 4 in size and had a 1-inch 16-bit CCD camera with 1024 × 1024 scanning lines. This camera generated 1200 raw images. Based on the obtained data, two-dimensional slice data were prepared using the back-projection method. Images were constructed via the volume-rendering method from slice data using 3D construction software (Imaris, Bitplane, Zürich, Switzerland) [20–22].

We measured the shortest distance from the center of the GPF as follows: the posterior end of the maxillary tuberosity, the medial surface of the maxillary tuberosity, and the anterior margin of the pterygoid hamulus (Fig. 3b, c). The thickness of the lateral and medial walls of the GPC was measured by dividing it into three portions (Fig. 3d-l). The inferior portion corresponds to the plane below the inferior nasal concha (INC) (Fig. 3d, g). The middle portion corresponds to the plane at the same level as the inferior margin of the INC (Fig. 3e, h). The superior portion corresponds to the plane at the same level as the connection site between the maxilla and the INC (Fig. 3f, i). All images were analyzed using Image-Pro 148 (Media Cybernetics, MD, Unites States).

All statistical analyses were conducted using EZR (Saitama Medical Center, Jichi Medical University, Saitama, Japan), a GUI for R (The R Foundation for Statistical Computing, Vienna, Austria). More precisely, it is a modified version of R Commander designed to incorporate frequently used statistical functions in biostatistics. The data were deemed statistically significant with P < 0.05.

## Results

### Anatomical analysis

**Fig. 1:**
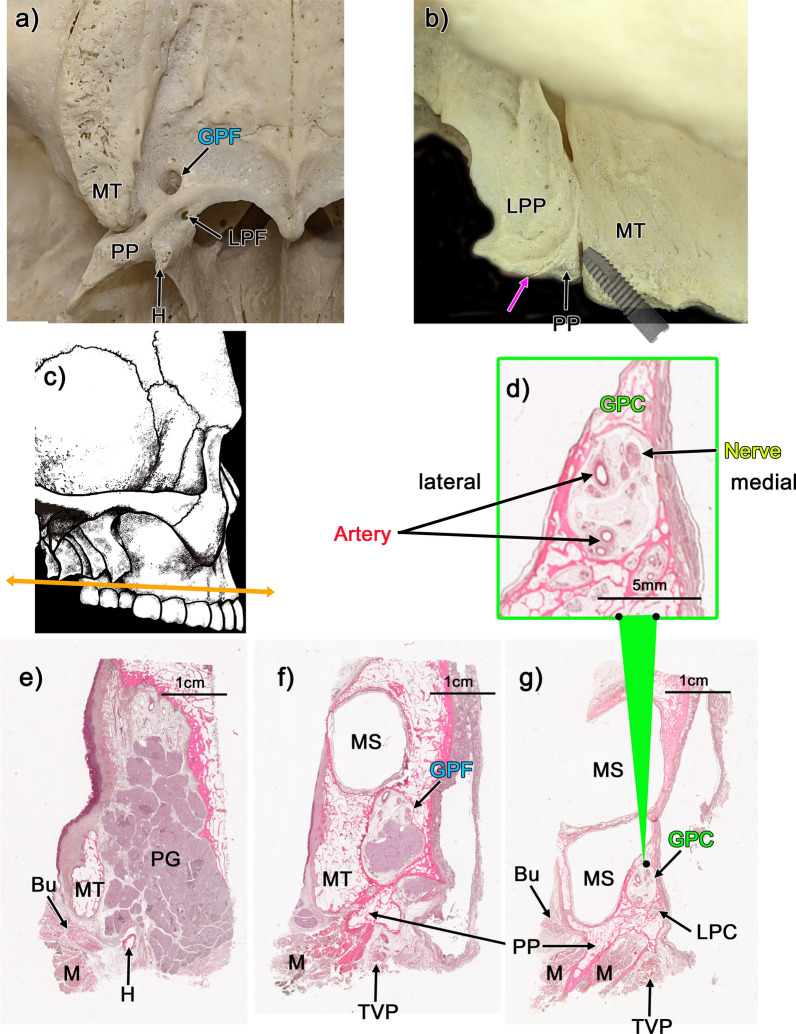
Greater palatine canal and its surrounding structures. **a** Inferior view of the greater palatine foramen (GPF) and its surrounding structures in a dry skull. **b** Lateral view of the dry skull and simulation of the pterygoid implant. An unnamed suture (pink arrow) was identified between the LPP and PB. Panel **c** shows a schematic illustration of the cross-sectional locations of **d-g**. **d–g**: Histological analysis of the GPF and its surrounding structures. All panels are horizontal sections. **e** (**g**) shows the most inferior (superior) side. **d** is a high-magnification view of panel **g**. The descending palatine artery is located lateral to the greater palatine nerve (**e**, **f**). Bu, buccinator muscle; M, medial pterygoid muscle; MS, maxillary sinus; GPC, greater palatine canal; GPF, greater palatine foramen; H, pterygoid hamulus; MT, maximally tuberosity; LPC, lesser palatine canal; LPF, lesser palatine foramen; LPP, lateral pterygoid process of the sphenoid; PP, pyramidal process of the palatine bone; TVP, tensor veli palatini muscle

**Fig. 2:**
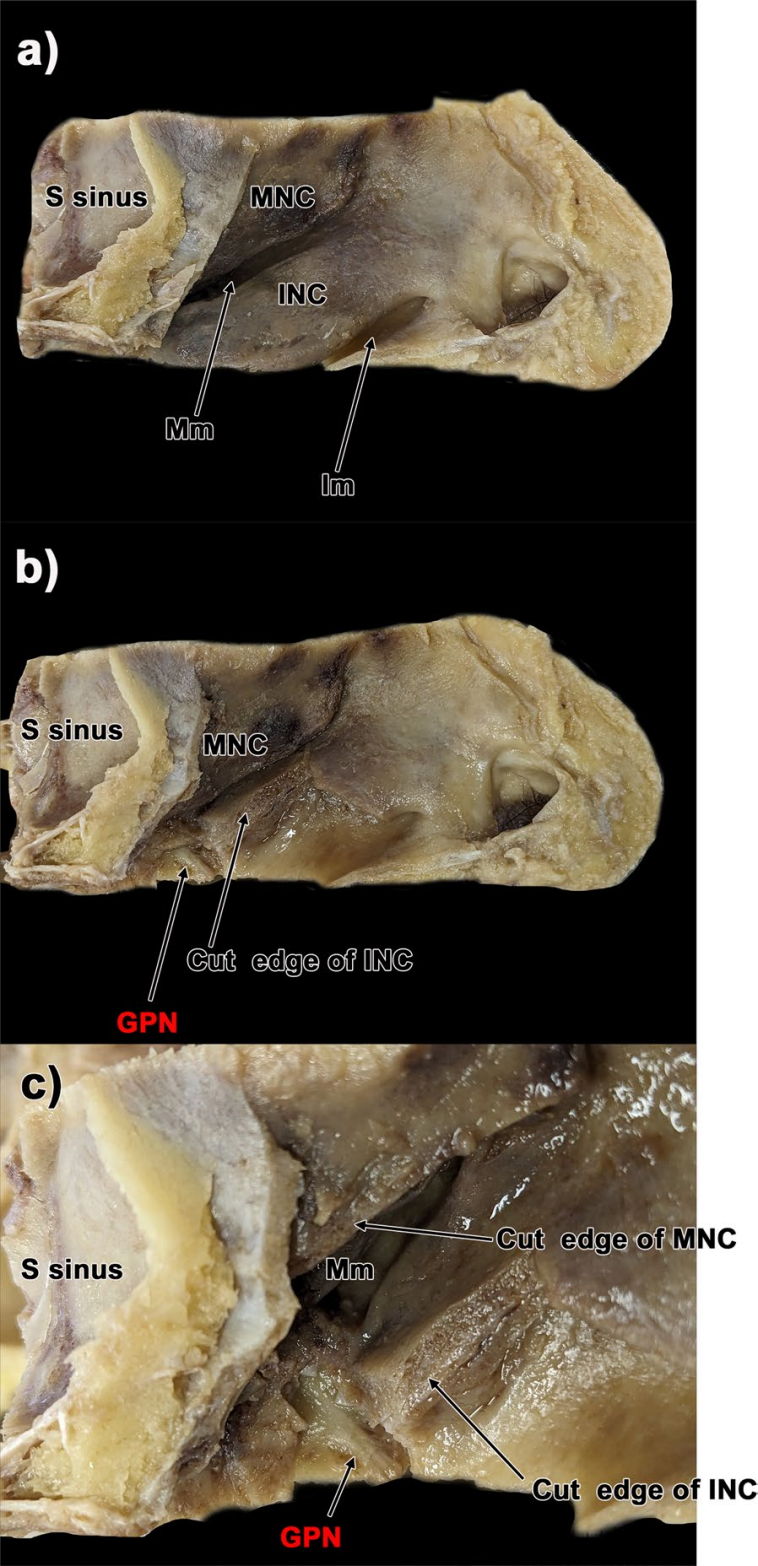
Gross anatomical analysis in the greater palatine canal. **a** Medial view of the nasal cavity before dissection. **b** Medial view of the nasal cavity after cutting the edge of the inferior nasal concha. **c** High-magnification view of **b** After removing a portion of the inferior nasal concha, only the greater palatine nerve was identified. The descending palatine artery was not observed. GPN: greater palatine nerve, Mm: middle nasal meatus, MNC: middle nasal concha, Im: inferior nasal meatus, INC: inferior nasal concha, S sinus: sphenoid sinus

The GPF or GPC was located anterior to the lesser palatine foramen or lesser palatine canal (LPC) (Fig. 1a, g), whereas the two canals were adjacent to the nasal cavity and maxillary sinus (Fig. 1g). The lateral view of the skull showed that the pyramidal process of the palatine bone was located between the maxillary tuberosity and the pterygoid process of the sphenoid bone (Fig. 1b). The unnamed suture appeared between the lateral pterygoid process and the pyramidal process (Fig. 1b). The low-magnification view of large histological sections (Fig. 1e–g) clarified that the greater palatine nerve always passed medial to the descending palatine artery in the GPC (10/10) (Fig. 1d, g). Gross anatomical analysis indicated that the greater palatine nerve was located lateral to the INC (Fig. 2a, b). However, the descending palatine artery was not identified after removing the medial wall of the GPC (Fig. 2c).

These results suggest that perforation of the GPC first damages the descending palatine artery rather than the greater palatine nerve.

### Micro-CT images

**Fig. 3:**
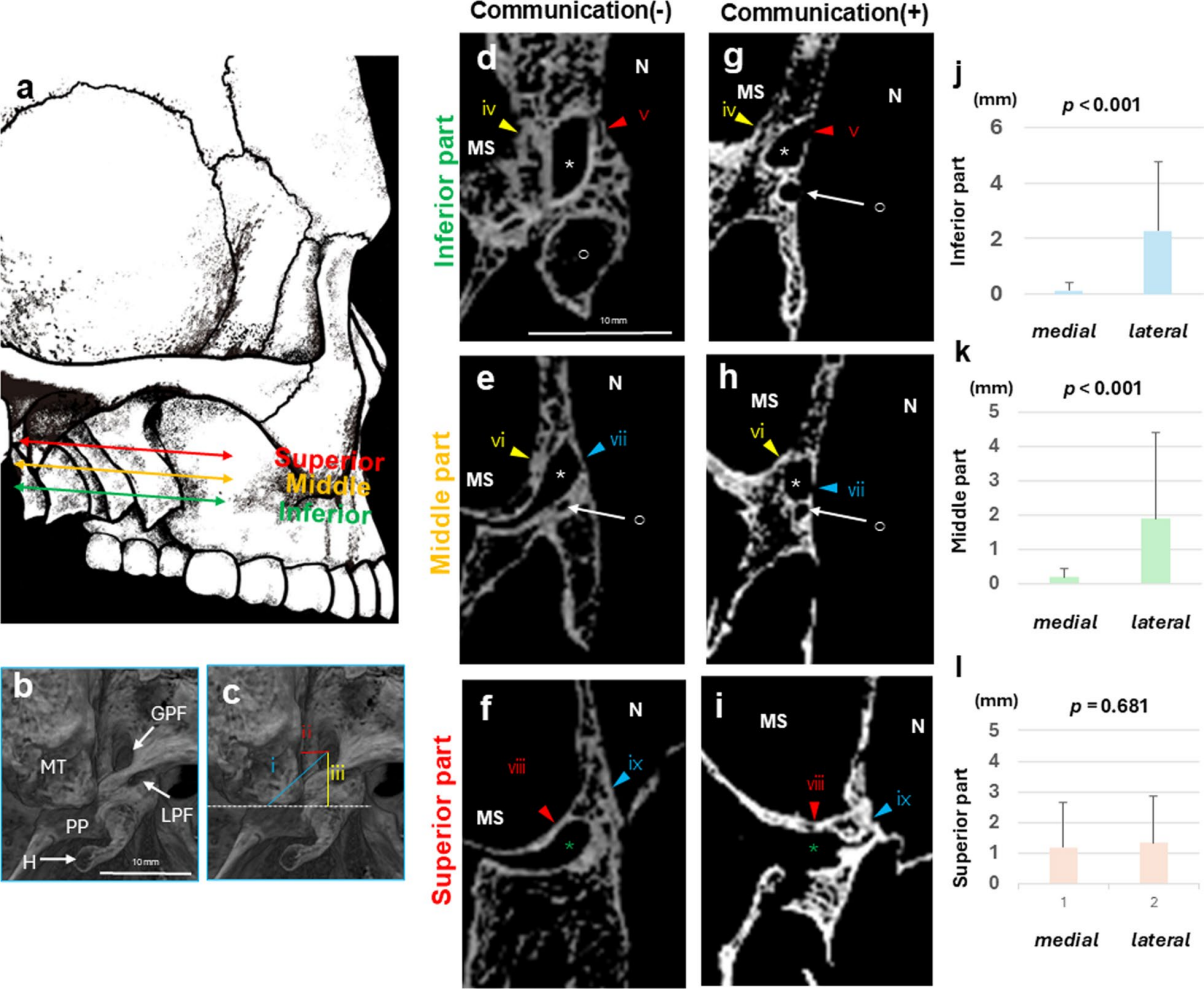
Micro-CT images of the greater palatine canal and its surrounding structures. **a** shows a schematic illustration of the cross-sectional locations of **d**–**i**. **b** and **c** depict an inferior view of the palate. **d**–**f**: Bone dehiscence on the medial bony wall of the GPC was not identified near the inferior nasal concha. **g**–**i**: Bone dehiscence was identified near the inferior nasal concha. **d** and **g** correspond to a plane below the inferior nasal concha (INC). **e** and **h** are planes at the same level as the inferior margin of the INC. Panels **f** and **i** are planes at the same level of the connection site between the maxilla and the INC. **g** and **h** show bone dehiscence. **j**–**l**: Comparison of the thickness between the medial and lateral walls of the greater palatine canals. GPF, greater palatine foramen; H, pterygoid hamulus; MT, maximally tuberosity; LPF, lesser palatine foramen; PP, pyramidal process of the palatine bone; Asterisk: greater palatine canal, Circle: lesser palatine canal, Green asterisk: fused palatine canal, N: nasal cavity, MS: maxillary sinus. The shortest distance from the center of the GPF (Panels b and c). i. GPF-Posterior end of maxillary tuberosity. ii. GPF-Medial surface of the maxillary tuberosity. iii. GPC-Anterior margin of the pterygoid Hamulus. The thickness of the lateral and medial walls of the GPC (Panels d-i). iv. The thickness of the lateral bony GPC. v. The thickness of the medial wall bony GPC. vi. The thickness of the lateral bony GPC. vii. The thickness of the medial wall bony GPC. viii. The thickness of the lateral bony GPC. ix. The thickness of the medial wall bony GPC

Micro-CT images also demonstrated that GPC passed between the nasal cavity and maxillary sinus (Fig. 3). The distance from the GPF and posterior end of the maxillary tuberosity was 11.44 ± 2.56 mm (Fig. 3a, e and Table 1). The shortest distance from the GPF and medial surface of the maxillary tuberosity was 4.28 ± 1.19 mm (Fig. 3a, e and Table 1). At the inferior level of the INC, the GPC ran parallel to the LPC (Fig. 3b, c, f, g). The fused palatine canal was identified at the same level as the connection site between the maxilla and the INC (Fig. 3d, h).

**Table 1:** Bone morphometry around the greater palatine canal

	i	ii	iii	iv	v	vi	vii	viii	ix
N	30	30	30	30	30	30	30	30	30
Mean ± SD (mm)	11.44 ± 2.56	4.28 ± 1.19	7.70 ± 2.24	2.28 ± 2.48	0.14 ± 0.27	1.89 ± 2.51	0.19 ± 0.26	1.34 ± 1.51	1.18 ± 1.46
Range (mm)	7.39–17.63	2.21–8.14	2.57–12.94	0.243–9.887	0–1.06	0–10.22	0—0.77	0–5.85	0–6.01

^a^GPF—Posterior end of maxillary tuberosity;

^b^GPF—Medial surface of the maxirally tuberosity

^c^GPC—Anterior margin of the pterygoid hamulus;

^d^The thickness of the lateral bony GPC

^e^The thickness of the medial wall bony GPC (below the INC)

^f^The thickness of the lateral bony GPC; g. The thickness of the medial wall bony GPC (at the same level as the inferior margin of the INC)

^h^The thickness of the lateral bony GPC; i. The thickness of the medial wall bony GPC (at the same level as the connection site between the maxilla and the INC)

Bone dehiscence on the medial bony wall of the GPC was identified near the INC in 72.4% of the cadavers (21/29) (Fig. 3g–i). The medial bony GPC was dehiscent in 69.0% of the cadavers (20/29) at the inferior portion (Fig. 3d, g) and 58.6% of the cadavers (17/29) at the middle portion (Fig. 3e, h). However, hardly any cadavers (6.9%, 2/29) showed medial wall dehiscence at the superior portion (Fig. 3f, i). In some cases (10.3%, 3/29), the lateral bony GPC was dehiscent, and the GPC was adjacent to the maxillary sinus (data not shown). The appearance of bone dehiscence in the GPC is described in Table 2.

**Table 2:** Appearance of bone dehiscence in the GPC

	Inferior part		Middle part		Superior part	
No	d (lateral)	e (medial)	f (lateral)	g (medial)	h (lateral)	i (medial)
1						
2						
3		〇		〇		
4		〇		〇		
5						
6		〇		〇		
7		〇				
8		〇	〇	〇		
9		〇		〇		
10		〇		〇		
11		〇		〇		
12						
13				〇	〇	〇
14		〇		〇		
15		〇				
16						
17		〇				
18		〇		〇		
19		〇	〇	〇	〇	〇
20		〇		〇		
21		〇		〇		
22		〇		〇		
23		〇		〇		
24						
25		〇		〇		
26		〇		〇		
27		〇				
28						
29						

^d^The lateral wall of the GPC

^e^The medial wall of the GPC (below the INC)

^f^The lateral wall of the GPC

^g^The medial wall of the GPC (at the same level as the inferior margin of the INC)

^h^The lateral wall of the GPC

^i^The medial wall of the GPC (at the same level as the connection site between the maxilla and the INC)

The bone thickness of the inferior and middle portions of the GPC differed significantly between the medial and lateral wall (*p* < 0.001; Fig. 3j, k). However, the thickness of the superior portion of the GPC (fused palatine canal) did not significantly differ between the medial and lateral wall (Fig. 3l). The thickness of the GPC wall is described in Table 1.

### Histological analysis of bone dehiscence in the GPC

**Fig. 4:**
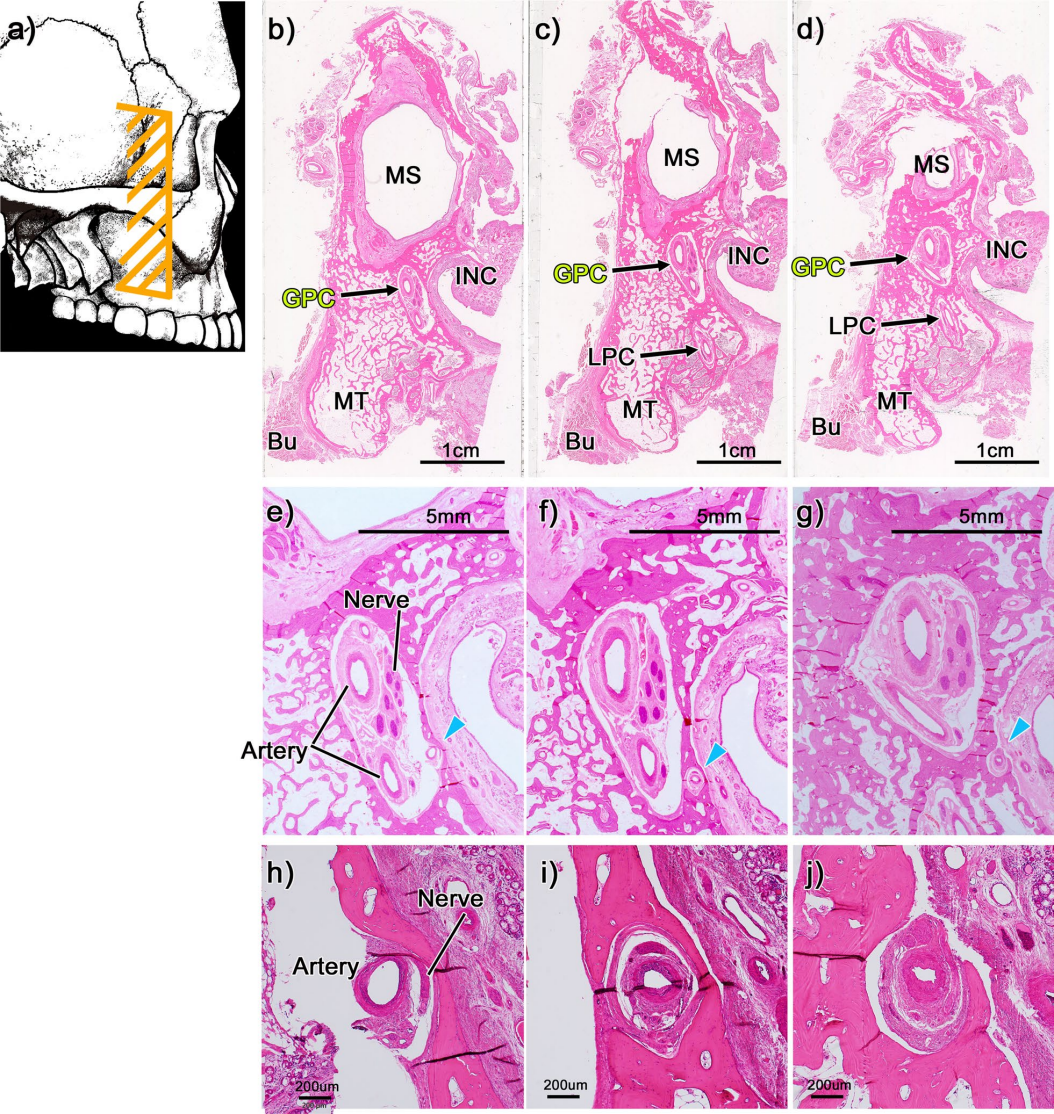
Histological analysis of bone dehiscence of the medial bony wall in the greater palatine canal. **a** shows a schematic illustration of the cross-sectional locations of panels **b**–**j**. All panels are frontal sections. **b** (**d**) shows the most anterior (posterior) side. **e**–**g** are high-magnification views of **b**–**d** respectively. **h**–**j** are high-magnification views of arrows of panels e–g respectively. The left-hand side corresponds to the lateral side. The greater palatine canal communicates with the nasal mucosa (**e**–**g**, arrowheads), and the artery and nerve pass through this continuation (**h**–**j**). BB, buccinator muscle; GPC, greater palatine canal; INC, inferior nasal concha; MS, maxillary sinus; MT, maximally tuberosity; LPC, lesser palatine canal

**Fig. 5:**
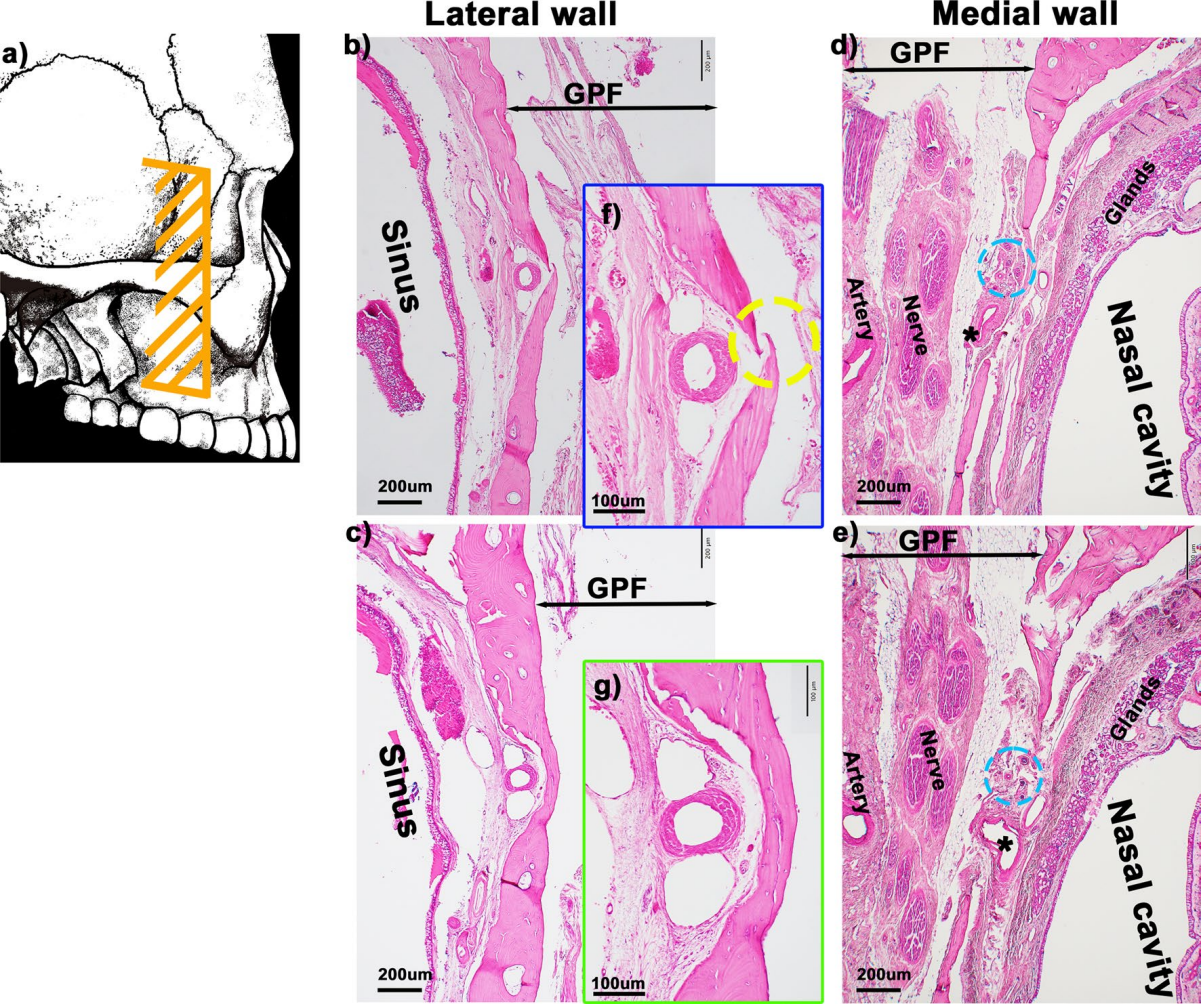
Histological analysis of bone dehiscence of medial and lateral bony walls in greater palatine canal. **a** shows a schematic illustration of the cross-sectional locations of panels **b-g**. **b-g** are frontal sections. Panels **b** and **c** show the lateral wall of the greater palatine canal (GPC). **d** and **e** show the medial wall of the GPC. **b** and **d** are anterior side compared with **c** and **e**. **f** and **g** are high-magnification views of panels **b** and **c**. The medial (**d** and **e**) and lateral (**f**) wall of the GPC have bone dehiscence. The nerve and artery passed through the bone dehiscence only at the medial wall (**d** and **e**). The yellow circle in panel **f** shows bone dehiscence at the lateral wall. The blue circles in **d** and **e** indicate nerves, whereas the asterisks in panels **d** and **e** indicate arteries. GPC, greater palatine canal, MS, maxirally sinus

In 50.0% of the cadavers (5/10), the GPC communicated with the nasal mucosa, with the artery and nerve passing through this continuation in the medial wall (Figs. 4e–j and 5d, e). Alternatively, one case (1/10) showed dehiscence in the lateral wall of the GPC but no passage of the artery and nerve through it (Fig. 5b, c).

## Discussion

Surgeons need to secure a safety space of 2 mm between the GPC and pterygoid implants [6]. Given the individual variations in the morphological characteristics of the posterior maxilla, pre-surgical assessment should be performed [10]. The most common complication (i.e., intraoperative bleeding) can probably be attributed to damage of the medial or lateral pterygoid muscles during the placement of a dental implant into the pterygoid process [1]. However, this bleeding can be stopped easily through local hemostasis measures and implant placement [23]. The descending palatine artery is thicker than the vessels around the pterygoid muscles. Our present study showed that the medial wall of the GPC communicated with the nasal cavity through the bone dehiscence through which the nerve and artery passed from the GPC to the nasal mucous membrane. Hence, perforation of the GPC can lead to nasal bleeding that is difficult to manage. Embedding the dental implant body into the posterior maxilla at an angle of 60° toward the lingual side has been found to increase the risk of damage to the GPC [24]. Therefore, surgeons must be careful not to place the dental implant body too close to the lingual side.

Traditional anatomical textbooks have described the nerves innervating the nasal mucous membranes [12, 13]. Most of the nasal branches enter the nasal cavity through the sphenopalatine foramen. However, a nasal branch is also given off by the greater palatine nerve. The lateral posterior inferior nasal (LPIN) nerve exits the canal through a small unnamed foramen on the vertical plate of the palatine bone and enters the nasal cavity. In the lateral wall of the nasal cavity, this nerve innervates the posterior inferior mucosa, including the INC. Ogi et al. [25] describe the anatomy of the pterygopalatine ganglion innervation of the lateral nasal wall. The same authors also mentioned that the greater palatine nerve gives off a branch through a small canal on the vertical plate of the palatine bone. Our results showed that a portion of the medial wall of the GPC opened to the nasal cavity near the INC. In addition, we found that the nerve and artery passed through this small canal. This nerve could possibly be the LPIN nerve, considering previous studies. However, no study has yet described whether the LPIN nerve passes with the artery. The sphenopalatine artery supplies the nasal cavity through the sphenopalatine foramen [12, 13], but the small artery did not pass through the foramen. Further studies are therefore needed to investigate this new artery passing through a tiny canal.

The GPF is an important structure for block anesthesia of the greater palatine nerve used during the treatment of multiple teeth in the posterior maxilla [26]. The location of the GPF in humans has been clarified mainly through micro-CT and dry skull studies. The majority of people have a GPF located adjacent to their third maxillary molars, whereas some have a GPF locate distal to the third molars [27, 28]. However, studies have shown racial variations in GPF location [29–36]. The length of the GPC is around 31.82 mm and can differ significantly according to sex. In fact, a systematic review and meta-analysis revealed that the mean length of the GPC was 26.97 mm, the angle between the vertical plane and axis of the GPC was 19.09°, and the angle between the transverse plane and the axis of the GPC was 62.63° [37]. Hence, perforation of the GPC by the pterygoid implants seems to spread the inflammation to the nasal cavity. Considering that previous studies have focused on bone morphometry for block anesthesia of the greater palatine nerve, further studies on pterygoid implants are needed.

## Conclusions

The present study demonstrated that the GPC communicated with the nasal cavity through bone dehiscence, which appeared near the INC in 72.4% of the cadavers (21/29). The nerve and artery passed from the GPC to the nasal mucous membrane through this dehiscence. Considering that the greater palatine nerve always passes medial to the descending palatine artery in the GPC, the descending palatine artery is first damaged rather than the greater palatine nerve during pterygoid implant surgery. Hence, dental surgeons who penetrate the GPC using an implant body may extend the bleeding to the nasal mucosa, which and the seems to spread the inflammation to the nasal cavity.

## Data Availability

The data sets used and analyzed during the current study are available from the corresponding author on reasonable request.

## Abbreviations

CT: Computed tomography
GPC: Greater palatine canal
GPF: Greater palatine foramen
INC: Inferior nasal concha
LPC: Lesser palatine canal
LPIN: Lateral posterior inferior nasal
